# The relationship of history of psychiatric and substance use disorders on risk of dementia among racial and ethnic groups in the United States

**DOI:** 10.3389/fpsyt.2023.1165262

**Published:** 2023-04-24

**Authors:** María P. Aranda, Jiaming Liang, Xinhui Wang, Lon S. Schneider, Helena C. Chui

**Affiliations:** ^1^Alzheimer’s Disease Research Center, University of Southern California, Los Angeles, CA, United States; ^2^USC Suzanne Dworak-Peck School of Social Work, Edward R. Roybal Institute on Aging, University of Southern California, Los Angeles, CA, United States; ^3^Keck School of Medicine, University of Southern California, Los Angeles, CA, United States

**Keywords:** psychiatric disorder, dementia subtypes, racial/ethnic disparities, survival analysis, substance use disorder

## Abstract

**Introduction:**

Dementia is characterized by significant declines in cognitive, physical, social, and behavioral functioning, and includes multiple subtypes that differ in etiology. There is limited evidence of the influence of psychiatric and substance use history on the risk of dementia subtypes among older underrepresented racial/ethnic minorities in the United States. Our study explored the role of psychiatric and substance use history on the risk of etiology-specific dementias: Alzheimer’s disease (AD) and vascular dementia (VaD), in the context of a racially and ethnically diverse sample based on national data.

**Methods:**

We conducted secondary data analyses based on the National Alzheimer’s Coordinating Center Uniform Data Set (N = 17,592) which is comprised a large, racially, and ethnically diverse cohort of adult research participants in the network of US Alzheimer Disease Research Centers (ADRCs). From 2005 to 2019, participants were assessed for history of five psychiatric and substance use disorders (depression, traumatic brain injury, other psychiatric disorders, alcohol use, and other substance use). Cox proportional hazard models were used to examine the influence of psychiatric and substance use history on the risk of AD and VaD subtypes, and the interactions between psychiatric and substance use history and race/ethnicity with adjustment for demographic and health-related factors.

**Results:**

In addition to other substance use, having any one type of psychiatric and substance use history increased the risk of developing AD by 22–51% and VaD by 22–53%. The risk of other psychiatric disorders on AD and VaD risk varied by race/ethnicity. For non-Hispanic White people, history of other psychiatric disorders increased AD risk by 27%, and VaD risk by 116%. For African Americans, AD risk increased by 28% and VaD risk increased by 108% when other psychiatric disorder history was present.

**Conclusion:**

The findings indicate that having psychiatric and substance use history increases the risk of developing AD and VaD in later life. Preventing the onset and recurrence of such disorders may prevent or delay the onset of AD and VaD dementia subtypes. Prevention efforts should pay particular attention to non-Hispanic White and African American older adults who have history of other psychiatric disorders.

Future research should address diagnostic shortcomings in the measurement of such disorders in ADRCs, especially with regard to diverse racial and ethnic groups.

## Introduction

Dementia is a significant public health problem and source of disability and mortality across the world. According to the WHO, it is estimated that over 55 million people live with dementia with nearly 10 million cases each year worldwide (1). A focus on etiological factors associated with dementia onset is crucial in the development of prevention, intervention, and supports, as well as setting research agendas for clinical care and public health programs. Based on data from a national registry representing dementia research and clinical trials in the United States (US), we investigated factors related to onset of two leading etiological dementia types, Alzheimer’s disease (AD) and Vascular Dementia (VaD). About 6.5 million adults in the US 65 years of age or older live with AD while 1.6 million live with VaD, and these estimates will grow significantly in the next three decades (2). Specifically, we draw attention to the role of various psychiatric and substance use disorders on incident dementia given the prevalence of such disorders in the US, and the fact that they are modifiable risk factors. It is estimated that 35.4 million (14.3%) US Americans reported psychiatric illness in the past year and 18.7 million had a substance use disorder while 8.5 million had both disorders (3). Identifying if specific racial and ethnic subgroups differ with regard to modifiable risk factors for incident dementia is key in tailoring dementia risk reduction efforts, especially for groups with documented high dementia rates (4–12). Thus, we address if race and ethnicity matter with regard to the association between various psychiatric and substance use disorders and incident dementia. Psychiatric and substance use disorders comprise often underappreciated categories of modifiable risk factors, and thus missed opportunities for elucidating pathways to dementia onset in the US (12–14). Understanding epidemiologic pathways to dementia sheds insights about risk and protective factors that may be pertinent to risk reduction and the attenuation of health disparities across all groups (15).

### Psychiatric disorders and dementia

Depression, the most widely cited psychiatric risk factor, is listed among the 12 preventable dementia risk factors in the 2020 Lancet dementia prevention report (1). Other psychiatric conditions, including anxiety (16, 17), schizophrenia (18), and bipolar disorders (19), are also linked to an increased risk of developing neurocognitive disorders later in life. Yet by far, most focus only on depression (20). According to Wingo and associates, psychiatric disorders include major depressive disorder, bipolar disorder, schizophrenia, anxiety disorders, post-traumatic stress disorder, and problematic alcohol use (although we treat this disorder separately in the next section). These disorders have up to four times higher risk for incident dementia in later life (13, 17, 18, 21–23) which may indicate a shared genetic and molecular basis for this link (24).

Previous work has reported associations between psychiatric conditions and different etiological subtypes of dementia. Anxiety was found to significantly increase the risk of developing AD and VaD by 50 to 80%, with chronic stress and inflammation cited as mechanistic conditions (16). A meta-analysis by Günak et al. (2020) found that PTSD is a significant risk factor for all-cause dementia, with a predicted increase of 61% in veterans and 111% in the general population (25). A study that followed Holocaust survivors found that VaD accounted for 60% of all-cause dementia caused by PTSD, while AD accounted for 20% (26). Schizophrenia has also been linked to an increased risk of dementia, particularly VaD, due to increased oxidative stress and decreased blood flow to the brain (27, 28). Bipolar disorder is associated with an increased risk of both AD and VaD, potentially due to the impact of manic episodes on the brain, as well as the use of medications that can affect brain function (19, 29).

### Substance disorders and dementia

Although limited alcohol intake in earlier adult life may be neuro-protective (30–32), previous work elucidates a link between harmful use of licit and illicit drugs and cognitive impairment (33). For instance, Xu and associates’ meta-analysis findings identified a dose–response associated with dementia between alcohol consumption and incident dementia with low and moderate alcohol consumption associated with reduced risk and excessive drinking, i.e.,10% increase risk with 23 drinks/week or ≥ 38/g/day (34). A recent review indicates that heavy alcohol use was associated with brain structure changes, cognitive impairments, and increased risk of all types of dementia (32).

In addition to alcohol use, excessive drug use has been found to lead to neuronal damage and increase risk of dementia. For instance, heavy smoking (>2 packs/day) and tobacco chewing are linked to a 79 and 78% increased risk of AD and VaD, respectively (30). A longitudinal study showed that heavy smoking in mid-life adults increased AD risk after age 60 by 150%, and the risk of VaD actually tripled (35). Illegal drug use can be more harmful to cognitive function. For example, amphetamine use was found to be associated with a 3.8-fold increased risk of AD and a 2.5-fold increased risk of VaD (36). Cocaine and heroin use can permanently alter tight junction protein expression and conformation in the brain, leading to neuroinflammation and increased VaD risk (37). The effects of other drugs, such as marijuana and cannabis, on cognitive function are still not conclusive: While some research found that weekly recreational marijuana users have lower memory performance compared to non-users, an increasing number of studies point to the potential benefits of medical cannabis in expanding blood vessels and protecting the nervous system (38, 39).

### Racial and ethnic considerations

There is limited evidence of the influence of psychiatric and substance use history on the risk of dementia subtypes among racial/ethnic minorities in the US. This is unfortunate given that studies examining racial and ethnic disparities in incident dementia have consistently found higher rates at least for African Americans, Hispanics, and others (4–12). In terms of incident dementia rates, African Americans (19 to 27 cases per 1,000) were found to have two to three times higher risk of developing dementia compared to their White counterparts (11 cases per 1,000) (10, 12). Similarly, Hispanics tend to have a 50% higher risk of developing dementia (12 to 19 cases per 1,000) than non-Hispanic White people (10). Other minority groups, such as American Indians/Alaska Natives, Asian Americans, and Pacific Islanders, have also been found to have higher rates of incident dementia compared to non-Hispanic White people, although the magnitude of this disparity may vary depending on the specific subgroup examined (5, 10). Several reasons for the incident dementia disparities have been implicated such as unequal access to and quality of care, comorbidities such as cardiovascular disease, differentials in socioeconomic status and lifestyle behaviors (physical activity, smoking, social engagement), and the health effects of racism and discrimination (5–10, 40, 41).

The situation regarding racial/ethnic disparities in the diagnostic rates of mental disorders is somewhat complex. On the one hand, research has observed the Black-White mental health paradox, in which African Americans have lower or similar rates of mental disorders relative to White people (42). For example, surveys on adolescents and adults in the US show that White people have the highest prevalence of depression and anxiety symptoms at 15.8 and 7% respectively, which is similar to Hispanics but higher than African Americans and other non-Hispanic races (43, 44). Even during the COVID-19 pandemic, Black people facing higher risks of income and food insecurity still reported significantly lower levels of perceived stress and worry than White people (39% vs. 25%) (45). There is evidence that further confirms the mental health advantages of Black people relative to White people were widened after adjusting for socioeconomic factors (42). On the other hand, some other studies reported that racial/ethnic minorities, including African Americans, were more susceptible to mood disorders, like depression and anxiety, than non-Hispanic White people after adjusting for factors such as socioeconomic status, medical treatment resources, and attitudes toward the medical system (45–47). Such disparities were more pronounced among people with low education (48).

In contrast to depression and anxiety, studies on racial/ethnic disparities in psychotic disorders have been relatively consistent. Racial/ethnic minorities, including African Americans, Hispanics, Asians, and Native Americans, showed higher diagnostic rates of severe—psychosis related—mental illnesses than non-Hispanic White people (49, 50). For example, African Americans have a 5-time higher risk of developing schizophrenia than non-Hispanic White people, while Hispanics have a nearly 3 times higher risk (51).

Turning to alcohol use, national estimates of heavy alcohol use indicate that the highest rate is among younger aged adults: 18 to 25 (7.1%), followed by adults aged 26 or older (6.3%). With regard to race/ethnicity, non-Hispanic White people were more likely to be heavy alcohol users in the past month compared with African Americans, 6.7% vs. 5.2%, respectively. Hispanics and Asian Americans reported lower rates at 4.7 and 1.9%, respectively.

Beyond alcohol consumption rates, we turn our attention to differential rates of substance use disorders (3). According to the 2021 National Survey on Drug Use and Health, substance use (as well as psychiatric) disorders “are characterized by impairment caused by the recurrent use of alcohol or other drugs (or both), including health problems, disability, and failure to meet major responsibilities at work, school, or home” (3). Specifically, the report identifies that when we consider substance use disorders we see differential rates with regard to race/ethnicity such that American Indian and Alaska Native people report the highest rates (27.6%) followed by multiracial people (25.9%) compared to African Americans and White people who have similar rates, 17.22 and 17%, respectively. Hispanics report 15.7%, and Asian Americans have the lowest rate at 8%. Thus, the racial/ethnic picture in terms of alcohol and other substance use rates is a bit more complex.

In sum, our study explores the role of psychiatric and substance use history on the risk of etiology-specific dementias: AD and VaD, in the context of a large, racially and ethnically diverse cohort of adult research participants in the network of US Alzheimer Disease Research Centers. Based on survival analyses, we characterize the cognitively intact sample with regard to incident AD and VaD rates, rates of psychiatric and substance use disorders, and the risk of incident dementia by race and ethnicity, specifically non-Hispanic White people, African Americans, Asian Americans, and other race/ethnicity. Our findings are poised to inform future risk reduction targets to delay or avoid the onset of dementia in the US population.

## Materials and methods

### Study sample

This study used data from the Uniform Data Set (UDS) of the National Alzheimer’s Coordinating Center (NACC). The NACC was established in 1999 to facilitate collaborative research among the NIA-funded Alzheimer’s Disease Research Centers (ADRCs) in the US and to develop and maintain a large, standardized longitudinal clinical and neuropathological research database, titled the UDS. Since 2005, the UDS has collected annual visit information from participants, including demographic characteristics, clinical and neurological examination findings, diagnosis, etc. Dementia diagnosis is made by either a consensus team or a single physician (the one who conducts the examination). Each ADRC has its own IRB-approved protocol for recruiting participants, and written informed consent from all participants and co-participants was obtained. Therefore, the use of publicly available UDS data in this study is exempt from IRB. The collection of NACC data is based on clinical referral and voluntary participation, not a population-based sample of the US population (52).

This study was based on UDS data from 2005 to 2019. Study participants were those who (1) participated in at least 2 visits, (2) were dementia-free at baseline, and (3) had a history of psychiatric disorders assessed on at least one visit. To examine the effect of history of psychiatric disorders on risk of dementia in different race/ethnicity groups, participants with missing values on race/ethnicity were excluded (n = 179). The final analytic sample contained 17,592 participants.

### Measurements

#### Etiological subtypes of dementia: AD and VaD

Dementia diagnoses are derived from clinicians or consensus conferences based on structured clinical records, neuropsychological test findings, and relevant symptom and functional assessments (53). Participants were identified in four categories: normal, impaired not MCI (mild cognitive impairment), MCI, and dementia. The etiological diagnoses were based on the National Institute of Neurological and Communicative Disorders and Stroke/Alzheimer Disease and Related Disorders Association (NINCDS-ADRDA) criteria prior to 2015 and according to National Institute on Aging/Alzheimer’s Association (NIA-AA) criteria from 2015 onward, when the UDS version 3 was updated (54, 55). The data encompass 33 diverse dementia etiologies, such as AD, VaD, Lewy body disease, frontotemporal dementia, and corticobasal degeneration, etc. Given the possibility of mixed-etiology dementias among some participants, this study was limited to individuals whose primary etiology was AD or VaD.

#### History of psychiatric and substance use disorders

Depression history is reflected by two items: (1) if the participant has active depression for the last 2 years; and (2) if the participant had depressive episodes more than 2 years ago. Both items are binary, and “yes” on any item would be recorded as having depression history.

In terms of traumatic brain injury (TBI) history, three items ask if the participant had TBI (1) without loss of consciousness, (2) with <5 min loss of consciousness, and (3) with ≥5 min loss of consciousness. An endorsement of any item indicates having a TBI history.

History of other psychiatric disorders combined other psychiatric disorders except for depression, including post-traumatic stress disorder, bipolar disorder, schizophrenia, anxiety, obsessive-compulsory disorder, and developmental neuropsychiatric disorder. The questionnaire employed in the study has undergone revision, evolving from earlier versions (1.2–2.0) that only utilized a single variable, “other psychiatric disorders,” to version 3.0, where each clinical diagnosis is documented separately. Thus, this study adopted the combined variable from earlier versions in order to avoid a reduction in sample size.

History of problematic alcohol use is reflected by an item that asks whether drinking has caused clinically significant impairment in areas like work, driving, legal, or social activities in the past 12 months.

Similarly, other substance use history is obtained by asking if participants report clinical impairment in areas like work, driving, legal, or social activities due to use of other substances in the past 12 months. If answered yes, a subsequent query will prompt participants to specify the name of the abused substance. The most frequently reported answers include marijuana, cannabis, and cocaine, among others.

We also generated a summary variable to indicate if participants had a history of at least one psychiatric or substance use disorder listed above.

#### Race/ethnicity

Participants’ race and ethnicity were self-reported. For racial identity, if a participant reported multiple races, they would be asked about their primary, secondary, or tertiary racial identification. There is a single question asking about Hispanic ethnicity regardless of race. We derived a 5-category variable for racial/ethnic identity of participants, including 1-Hispanic, 2-non-Hispanic (NH) White person, 3-NH African American, 4-NH Asian American, and 5-NH Others. We combined American Indian or Alaska Native, Native Hawaiian or Other Pacific Islander, other specified races, and those with mixed race, as others due to their small sample sizes.

#### Other covariates

Other factors related to risk of incident dementia in older adults were considered in the analyses. Demographic characteristics include age (in years), sex (dichotomous biological sex of participants at birth, male/female), and level of education (≤high school/ >high school). Health-related and genetic risk factors include body mass index (BMI), frontotemporal lobar degeneration (FTLD) mutation, and number of *APOE* e4 alleles. BMI was calculated from height and weight measurements. For cross-race/ethnicity comparisons, we categorized participants’ BMI according to WHO BMI classifications: 1-underweight (<18.5), 2-normal (18.5–24.9), 3-overweight (25–29.9), and 4-obese (≥30). A specific Asian BMI criteria was used for Asian participants (underweight: <18.5, normal: 18.5–22.9, overweight: 23–24.9, and obese: ≥25) (56). The NACC has an independent FTLD Module that incorporates family history, neuropsychological symptoms, and medical and imaging evidence to assess whether participants are carriers of FTLD-related mutations. From these, we generated a variable to indicate whether participants have a hereditary FTLD mutation (57). The e4 variant of *APOE* genotype is considered a genetic risk factor for AD and related dementias. The NACC reports the number of *APOE* e4 alleles as 0, 1, and 2 (58).

### Analyses

Descriptive statistics were used to summarize the baseline characteristics of participants. Continuous variables were presented with mean and standard deviation, and categorical variables were presented with counts and percentages. Bivariate correlational analyses were conducted to (1) select covariates to be included in cox proportional hazard models and (2) assess potential risk of multi-collinearity.

We then conducted survival analyses to investigate variables related to increased risk of AD and VaD during follow-up using Cox proportional hazard modeling. Survival analysis is a commonly used longitudinal analytic approach which measures the time to an event or outcome of interest. An event in this study was defined as the onset of AD and VaD (incident dementia) during follow-up, and censoring at the last date of contact was used to account for participants who had no dementia before loss to follow-up or death (59). We chose not to employ competing risk modeling to account for drop-out due to death and the onset of other etiologies, because it assumes that events such as dementia onset and death are completely independent, which may not necessarily hold true in all cases. Also, given that the diagnosis of dementia and the collection of some medical and genetic data may not be independent across ADRC sites, it was necessary to adjust for ADRC-related random effects. Competing risk modeling, however, does not allow for such adjustments. Therefore, we fitted separate models for each etiological dementia (AD and VaD), treating other failure types as censored data (60). The Stata “shared[]” function was utilized to adjust the ADRC-related random effects in Cox proportional hazard models (61). Sensitivity analyses were conducted to compare the model fits between competing risk models that treat death as a competing risk, and Cox hazard models that adjust ADRC random effects. The results (i.e., c-index, AIC, BIC) demonstrated that the Cox models provided a better explanation of AD and VaD risks, and had superior goodness-of-fit (see Supplementary File 1).

The first set of six models estimated the relationship between each history of psychiatric and substance use disorder (i.e., any psychiatric and substance use disorder, depression, TBI, other psychiatric disorders, alcohol use, and other substance use) and the risk of AD. Then, an interaction term between history of psychiatric and substance use disorder and participants’ race/ethnicity was introduced in each model. If a significant interaction was found, then we estimated race/ethnicity-stratified hazard ratio (HR) for that psychiatric disorder. The same approach was used to estimate the second set of models on the relationship between psychiatric and substance use history and risk of VaD.

In AD models, the number of *APOE*-e4 alleles has a significant amount of non-random missingness (15%), so a multiple imputation approach was used to maximize the utilization of available data in the analyses. We performed multiple imputations by chained equations (MICE) to estimate the missing data of *APOE*-e4 alleles. Age, gender, race/ethnicity, BMI, and dementia event were entered as predictors, because they either differed at baseline by missing status, or were related to missing patterns. To account for the skewed distribution of *APOE*-e4 alleles, it was suggested to generate at least 10 imputed datasets. In this study, the number of imputed datasets was increased to 20 to ensure the loss of efficiency to be less than 5% (62). All statistical analyses employed an alpha level for statistical significance of 0.05 (two-tailed) and were performed using Stata 15 (63).

## Results

### Participant characteristics

The analyses included a total of 17,592 participants recruited from 33 ADRCs and 4 Exploratory Centers across the US who were cognitively intact at baseline. During the 14-year data range, 3,253 (18.5%) people developed dementia, of whom 2,629 (14.94%) had AD as primary etiological cause with an average of 3 (± 2.30) years of follow-up, and 312 (1.77%) had VaD as etiological cause with an average of 3.4 (± 2.60) years of follow-up. The average follow-up period for cognitively normal participants was 4.9 (± 3.50) years. Table 1 summarizes participants’ demographics, health and genetic factors, and prevalence of psychiatric and substance disorders history by dementia status. The average age at baseline for the total sample was 71.5 (± 9.90) years with a range from 55 to 104, while nearly 60% self-identified as female, and 20% had high school education or less. Non-Hispanic White persons comprised three-quarters of the participants, followed by African Americans (12.9%), Hispanics (6.9%), and Asians (2.6%). 34% of participants had a BMI within the normal range, 39% were overweight, and 25% were obese. 27% of participants were identified as carriers of FTLD mutation, and about 35% had *APOE*-e4 alleles. The prevalence of psychiatric and substance use history were the following: depression (30.19%), TBI (11.75%), other psychiatric disorders (4.87%), alcohol use (4.06%), and other substance use (1.31%).

**Table 1:** Descriptive characteristics of participants at baseline according to development of etiological subtypes of dementia during follow-up.

		Total (*N* = 17,592)	Normal (*N* = 14,339)	AD^a^ (*N* = 2,629)		VaD^b^ (*N* = 312)	
		*N* (%)/Mean (SD)	*N* (%) / Mean (SD)	*N* (%) / Mean (SD)		*N* (%)/Mean (SD)	
*Demographics*							
Age at baseline (years)		71.5 (9.90)	70.41 (10.07)	73.48 (8.41)	***	74.21 (8.34)	***
Female		10,509 (59.70%)	9,039 (63.04%)	1,294 (49.22%)	***	157 (50.32%)	***
Education (≤ High school)		3,539 (20.12%)	2,713 (18.92%)	547 (20.18%)	*	79 (25.32%)	**
Race/Ethnicity	Hispanic	1,212 (6.90%)	1,028 (7.17%)	182 (6.92%)	***	22 (7.05%)	***
	Non-Hispanic White person	13,144 (74.70%)	10,529 (73.43%)	2026 (77.06%)		191 (61.22%)	
	African American	2,265 (12.90%)	1987 (13.86%)	263 (10.00%)		63 (20.19%)	
	Asian	451 (2.60%)	360 (2.51%)	80 (3.04%)		18 (5.77%)	
	Others	520 (3.00%)	434 (3.03%)	78 (2.97%)		18 (5.77%)	
*Health and Genetic Factors*							
BMI^c^	Underweight (BMI range?)	188 (1.07%)	141 (0.98%)	35 (1.33%)	***	3 (0.96%)	**
	Normal	6,039 (34.33%)	4,762 (33.21%)	966 (36.74%)		75 (24.04%)	
	Overweight	6,886 (39.14%)	5,553 (38.73%)	1,055 (40.13%)		121 (38.78%)	
	Obese	4,479 (25.46%)	3,882 (27.07%)	573 (21.80%)		113 (36.22%)	
FTLD mutation (yes)		47 (27.00%)	27 (0.19%)	5 (0.19%)		0 (0.00%)	
*APOE* e4 alleles	No e4	11,475 (65.20%)	9,957 (69.44%)	1,333 (50.69%)	***	208 (66.81%)	
	1 copy	5,351 (30.40%)	3,976 (27.73%)	1,024 (38.94%)		91 (29.31%)	
	2 copies	776 (4.40%)	406 (2.83%)	273 (10.37%)		12 (3.88%)	
*Psychiatric history*							
History of Any psychiatric and substance use disorders		7,097 (40.34%)	5,483 (38.24%)	1,217 (46.29%)	***	163 (52.24%)	***
Depression		5,311 (30.19%)	4,016 (28.01%)	922 (35.07%)	***	123 (39.42%)	***
Other psychiatric disorders^d^		856 (4.87%)	658 (4.59%)	168 (6.39%)	***	18 (5.77%)	
Traumatic brain injury (TBI)		2067 (11.75%)	1,649 (11.50%)	381 (14.49%)	***	37 (11.86%)	
Alcohol abuse		715 (4.06%)	548 (3.82%)	124 (4.72%)	*	21 (6.73%)	**
Other substance abuse		230 (1.31%)	204 (1.42%)	34 (1.29%)		7 (2.24%)	

^a^Alzheimer’s Disease; ^b^Vascular Dementia; ^c^Body Mass Index; ^d^Other psychiatric disorders include post-traumatic stress disorder (PTSD), bipolar disorder, schizophrenia, anxiety, obsessive–compulsive disorder (OCD), and developmental neuropsychiatric disorders; Bivariate comparisons were conducted between cognitive normal group and each dementia subtype group. *T*-test for continuous and *χ*2 test for categorical variables. *p < 0.05, **p < 0.01, ***p < 0.001.

We compared the distribution of each variable between participants with normal cognition and those who developed AD and VaD. Both AD and VaD participants were older at baseline, had a lower proportion of females, and had lower education levels compared with the cognitively normal participants. In addition, AD participants were more likely to be non-Hispanic White people, non-obese, and have *APOE*-e4 alleles, while the VaD group was more likely to be comprised racial/ethnic minorities, and obese participants. For history of psychiatric and substance use disorders, compared to the cognitive normal group, people with AD had higher prevalence of depression, other psychiatric disorders, TBI, and alcohol use. The VaD group had higher prevalence of depression and alcohol use only.

Bivariate correlations showed that the onset of AD was significantly associated with all covariates, while the onset of VaD was not associated with FTLD mutation and number of *APOE*-e4 alleles. Thus, the AD models were adjusted for all covariates and the VaD models do not include FTLD mutation and *APOE*-e4 alleles. Variance inflation factors of all covariates were estimated and none is greater than 5 indicating multicollinearity is not present in our data.

### Estimating the risk of AD and VaD

We fitted a set of multivariable-adjusted Cox regression models to analyze the associations between history of psychiatric and substance use disorders and the risk of AD onset (Table 2). Model 1.1 found that having any history of psychiatric and substance use disorders increased the hazard of developing AD by 45% (HR: 1.45, 95% confidence interval [CI]: 1.33–1.57). Models 1.2–1.5 revealed the risk of each psychiatric and substance use disorder history on AD onset: depression (HR: 1.51, 95%CI: 1.39–1.64), other psychiatric disorders (HR: 1.25, 95%CI: 1.12–1.40), TBI (HR: 1.32, 95%CI: 1.13–1.55), and alcohol abuse (HR: 1.22, 95%CI: 1.02–1.47). No significant effect of other substance use was found on the risk of AD onset (Model 1.6, HR: 1.27, 95%CI: 0.90–1.79).

**Table 2:** Cox proportional hazard models for risk of AD (Alzheimer’s disease) onset.

		Model 1.1	Model 1.2	Model 1.3	Model 1.4	Model 1.5	Model 1.6
		HR (95% CI)	HR (95% CI)	HR (95% CI)	HR (95% CI)	HR (95% CI)	HR (95% CI)
History of any psychiatric or substance use disorders		1.45 (1.33, 1.57)***					
Depression			1.51 (1.39, 1.64)***				
Other psychiatric disorders^a^				1.25 (1.12, 1.40)***			
Traumatic brain injury (TBI)					1.32 (1.13, 1.55)**		
Alcohol abuse						1.22 (1.02, 1.47)*	
Other substance abuse							1.27 (0.90, 1.79)
Race/Ethnicity (ref: non-Hispanic White person)							
	Hispanic	1.27 (1.07, 1.50)**	1.26 (1.06, 1.49)**	1.26 (1.07, 1.50)**	1.27 (1.07, 1.50)**	1.26 (1.06, 1.49)**	1.26 (1.06, 1.50)**
	African American	0.97 (0.84, 1.12)	0.98 (0.85, 1.13)	0.93 (0.80, 1.07)	0.92 (0.80, 1.06)	0.92 (0.79, 1.06)	0.91 (0.79, 1.05)
	Asian	1.19 (0.94, 1.51)	1.17 (0.93, 1.48)	1.12 (0.89, 1.42)	1.11 (0.88, 1.40)	1.11 (0.88, 1.40)	1.11 (0.88, 1.40)
	Others	1.32 (1.05, 1.67)*	1.32 (1.04, 1.66)*	1.28 (1.02, 1.62)*	1.28 (1.01, 1.61)*	1.28 (1.01, 1.62)*	1.28 (1.02, 1.62)*
Age		1.04 (1.04, 1.05)***	1.04 (1.04, 1.05)***	1.04 (1.04, 1.05)***	1.04 (1.04, 1.05)***	1.04 (1.04, 1.05)***	1.04 (1.04, 1.05)***
Female (ref: male)		0.65 (0.60, 0.70)***	0.63 (0.58, 0.68)***	0.66 (0.61, 0.71)***	0.65 (0.60, 0.70)***	0.65 (0.6, 0.71)***	0.65 (0.6, 0.71)***
Education (ref: > high school)		1.21 (1.09, 1.34)***	1.21 (1.10, 1.34)***	1.21 (1.09, 1.34)***	1.20 (1.09, 1.33)***	1.2 (1.08, 1.33)***	1.20 (1.09, 1.33)***
BMI^b^ (ref: normal)							
	Underweight	1.13 (0.80, 1.59)	1.14 (0.81, 1.60)	1.17 (0.84, 1.65)	1.17 (0.83, 1.65)	1.18 (0.84, 1.66)	1.18 (0.84, 1.65)
	Overweight	0.89 (0.81, 0.97)*	0.89 (0.82, 0.98)*	0.89 (0.82, 0.98)*	0.90 (0.83, 0.99)*	0.90 (0.82, 0.98)*	0.88 (0.82, 0.98)*
	Obese	0.83 (0.74, 0.92)**	0.82 (0.74, 0.92)***	0.84 (0.75, 0.93)**	0.85 (0.76, 0.94)**	0.84 (0.76, 0.94)**	0.84 (0.76, 0.94)**
FTD mutation		1.04 (0.43, 2.51)	1.09 (0.45, 2.62)	1.00 (0.41, 2.41)	0.98 (0.40, 2.36)	0.99 (0.41, 2.39)	1.00 (0.42, 2.43)
*APOE* e4 alleles (ref: no e4)							
	1 copy	1.70 (1.55, 1.86)***	1.69 (1.54, 1.86)***	1.71 (1.56, 1.87)***	1.70 (1.55, 1.87)***	1.70 (1.55, 1.86)***	1.70 (1.55, 1.87)***
	2 copies	3.51 (3.02, 4.07)***	3.49 (3.01, 4.06)***	3.54 (3.05, 4.12)***	3.53 (3.04, 4.11)***	3.55 (3.05, 4.12)***	3.55 (3.05, 4.13)***
Latent random effects of ADC		*x̄*^2^ = 763.95***	*x̄*^2^ = 778.71***	*x̄*^2^ = 777.76***	*x̄*^2^ = 785.15***	*x̄*^2^ = 790.15***	*x̄*^2^ = 789.42***

^a^Other psychiatric disorders include post-traumatic stress disorder (PTSD), bipolar disorder, schizophrenia, anxiety, obsessive–compulsive disorder (OCD), and developmental neuropsychiatric disorders; ^b^Body Mass Index. *p < 0.05, **p < 0.01, ***p < 0.001.

For covariates, across the six models, Hispanic participants (e.g., in Model 1.1, HR: 1.27, 95%CI: 1.07–1.50) and participants in the other racial/ethnic group category (e.g., in Model 1.1, HR: 1.32, 95%CI: 1.05–1.67) tended to have increased hazards of developing AD than their non-Hispanic White counterparts. Older age, male gender, lower education level, and more *APOE*-e4 alleles was also associated with higher AD onset risk. Finally, compared to participants with normal BMI, overweight (e.g., in Model 1.1, HR: 0.89, 95%CI: 0.81–0.97), and obese (e.g., in Model 1.1, HR: 0.83, 95%CI: 0.74–0.92), participants showed lower risk of AD onset. Significant latent random effects under each model indicated that the implementation standard (e.g., data collection and dementia diagnosis) may slightly differ across ADRCs, confirming the necessity of adjusting cross-ADRC random effects.

The same analytical strategy was applied to study the risk of VaD onset (Table 3). Model 2.1 indicates that having any history of psychiatric and substance use disorders increased the hazard of developing VaD by 46% (HR: 1.46, 95%CI: 1.35–1.58). Models 2.2–2.5 revealed the association of each psychiatric and substance use disorder history on VaD risk: depression (HR: 1.53, 95%CI: 1.41–1.66), other psychiatric disorders (HR: 1.24, 95%CI: 1.11–1.38), TBI (HR: 1.33, 95%CI: 1.14–1.56), and alcohol use (HR: 1.22, 95%CI: 1.02–1.46). No significant effect of other substance use was found on the risk of developing VaD (Model 2.6, HR: 1.19, 95%CI: 0.84–1.67).

**Table 3:** Cox proportional hazard models for risk of VaD (Vascular Dementia) onset.

		Model 2.1	Model 2.2	Model 2.3	Model 2.4	Model 2.5	Model 2.6
		HR (95% CI)	HR (95% CI)	HR (95% CI)	HR (95% CI)	HR (95% CI)	HR (95% CI)
History of any psychiatric or substance use disorders		1.46 (1.35, 1.58)***					
Depression			1.53 (1.41, 1.66)***				
Other psychiatric disorders^a^				1.24 (1.11, 1.38)***			
Traumatic brain injury (TBI)					1.33 (1.14, 1.56)***		
Alcohol abuse						1.22 (1.02, 1.46)*	
Other substance abuse							1.19 (0.84, 1.67)
Race/Ethnicity (ref: non-Hispanic White person)							
	Hispanic	1.20 (1.01, 1.42)*	1.19 (1.00, 1.41)*	1.20 (1.01, 1.42)*	1.20 (1.01, 1.42)*	1.19 (1.00, 1.41)*	1.19 (1.00, 1.41)*
	African American	1.01 (0.88, 1.17)	1.02 (0.88, 1.17)	0.96 (0.84, 1.11)	0.96 (0.83, 1.10)	0.95 (0.83, 1.09)	0.95 (0.83, 1.09)
	Asian	1.05 (0.83, 1.33)	1.03 (0.82, 1.30)	0.99 (0.79, 1.25)	0.98 (0.78, 1.24)	0.98 (0.78, 1.24)	0.98 (0.77, 1.23)
	Others	1.37 (1.09, 1.73)**	1.37 (1.09, 1.73)**	1.33 (1.05, 1.67)*	1.32 (1.05, 1.67)*	1.32 (1.05, 1.67)*	1.33 (1.05, 1.67)*
Age		1.04 (1.03, 1.04)***	1.04 (1.03, 1.04)***	1.03 (1.03, 1.04)***	1.03 (1.03, 1.04)***	1.03 (1.03, 1.04)***	1.03 (1.03, 1.04)***
Female (ref: male)		0.63 (0.58, 0.68)***	0.61 (0.56, 0.66)***	0.64 (0.59, 0.69)***	0.63 (0.58, 0.68)***	0.64 (0.57, 0.69)***	0.63 (0.58, 0.68)***
Education (ref: >high school)		1.22 (1.10, 1.35)***	1.23 (1.11, 1.36)***	1.22 (1.11, 1.35)***	1.22 (1.10, 1.35)***	1.22 (1.09, 1.34)***	1.22 (1.10, 1.35)***
BMI^b^ (ref: normal)							
	Underweight	1.20 (0.86, 1.69)	1.22 (0.87, 1.71)	1.26 (0.89, 1.77)	1.25 (0.89, 1.76)	1.26 (0.89, 1.77)	1.26 (0.87, 1.77)
	Overweight	0.86 (0.79, 0.94)**	0.86 (0.79, 0.94)**	0.87 (0.79, 0.95)**	0.87 (0.80, 0.96)**	0.87 (0.79, 0.95)**	0.87 (0.79, 0.95)**
	Obese	0.77 (0.69, 0.86)***	0.76 (0.69, 0.85)***	0.78 (0.70, 0.87)***	0.79 (0.71, 0.88)***	0.76 (0.71, 0.87)***	0.79 (0.71, 0.87)***
Latent random effects of ADC		*x̄*^2^ = 837.37***	*x̄*^2^ = 850.02***	*x̄*^2^ = 853.70***	*x̄*^2^ = 858.87***	*x̄*^2^ = 865.97***	*x̄*^2^ = 864.78***

^a^Other psychiatric disorders include post-traumatic stress disorder (PTSD), bipolar disorder, schizophrenia, anxiety, obsessive–compulsive disorder (OCD), and developmental neuropsychiatric disorders; ^b^Body Mass Index. *p < 0.05, **p < 0.01, ***p < 0.001.

For covariates, across the six models, Hispanic ethnicity (e.g., in Model 2.1, HR: 1.20, 95%CI: 1.01–1.42) and participants with other racial/ethnic identities (e.g., in Model 2.1, HR: 1.37, 95%CI: 1.09–1.73) tend to have increased hazards of developing VaD than their non-Hispanic White counterparts. Older age, male gender, and lower education level were also associated with higher VaD onset risk. Finally, similar to AD models, overweight (e.g., in Model 2.1, HR: 0.86, 95%CI: 0.79–0.94) and obese (e.g., in Model 2.1, HR: 0.77, 95%CI: 0.69–0.86) participants had increased hazards developing VaD than that of group with normal BMI.

### Racial/ethnic differences in the risk of AD and VaD

To examine whether the associations between history of various psychiatric and substance use disorders and onset of AD and VaD differ by racial/ethnic groups, interaction terms were introduced into each Cox regression model. In both AD and VaD models, only the history of other psychiatric disorders was found to significantly interact with participants’ racial/ethnic identity (Likelihood-Ratio between model comparison *p* = 0.01 for AD model, *p* < 0.001 for VaD model). Specifically, a significant positive interaction was found between other psychiatric disorder and African American with non-Hispanic White people as the reference group (in AD model, p = 0.01; in VaD model, p < 0.001), suggesting that African Americans with history of other psychiatric disorders have increased hazards of developing AD and VaD than non-Hispanic White people with history of other psychiatric disorders. History of depression, TBI, alcohol use, and other substance use show similar effects on the onset risk of AD and VaD of participants in different racial/ethnic groups (all LR tests *p* > 0.05, see Supplementary File 2).

**Figure 1:**
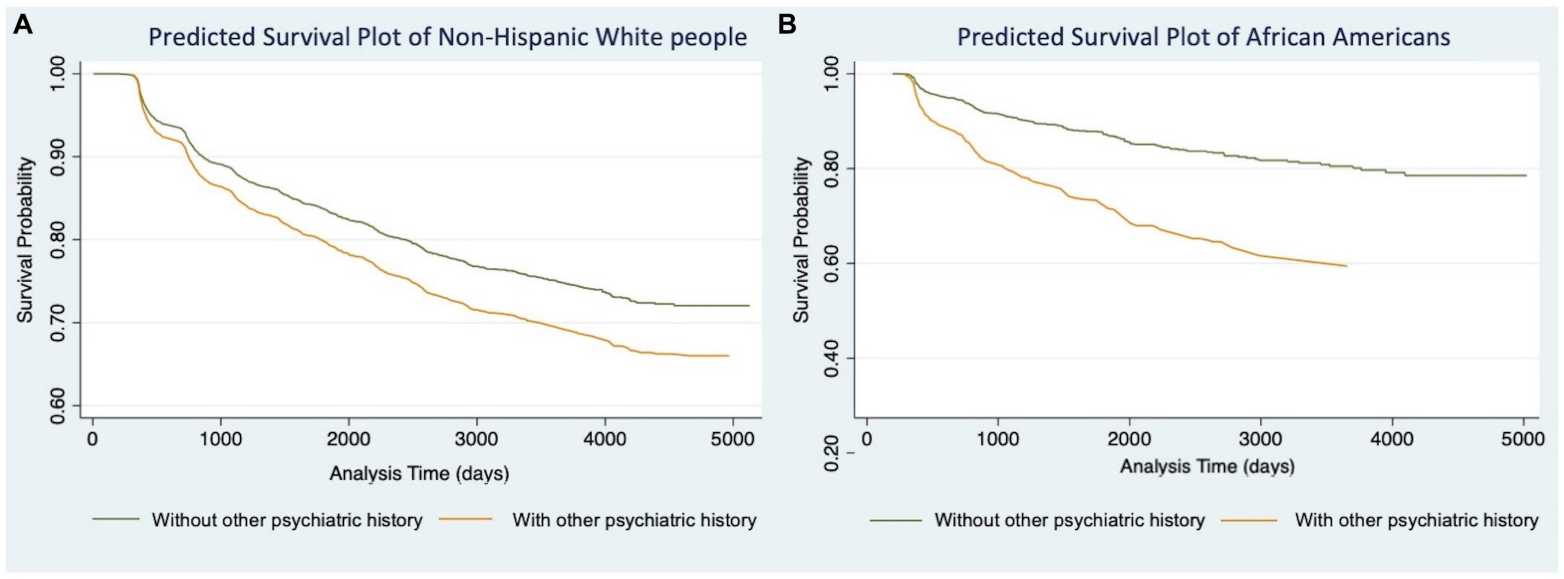
Cox proportional hazard models of Alzheimer’s Disease comparing estimated survival probability of both non-Hispanic White **(A)** and African American **(B)** older adults with or without history of other psychiatric disorders (i.e., post-traumatic stress disorder (PTSD), bipolar disorder, schizophrenia, anxiety, obsessive-compulsive disorder (OCD), and developmental neuropsychiatric disorders). Models were adjusted for participants’ age at baseline, gender, education level, BMI, FTD mutation, and number of *APOE*-e4 alleles. Results suggest that having history of other psychiatric disorders increases the risk of developing Alzheimer’s Disease.

**Figure 2:**
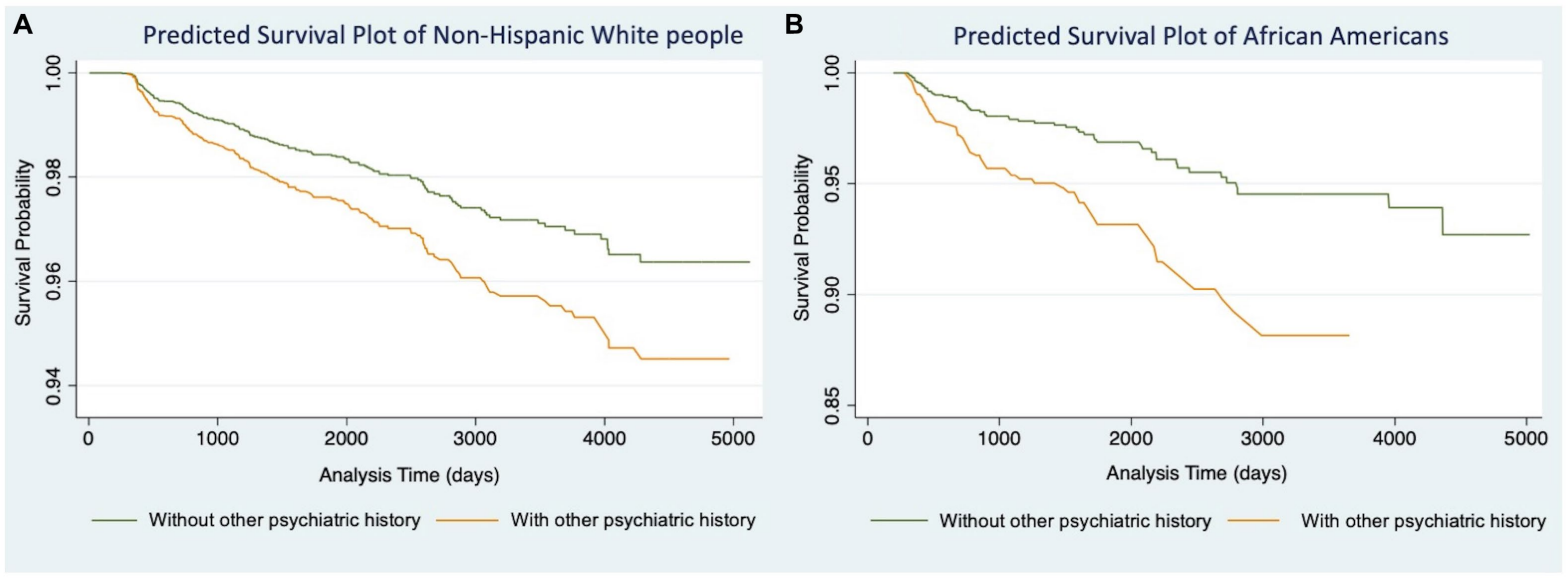
Cox proportional hazard models of Vascular Dementia comparing estimated survival probability of both non-Hispanic White **(A)** and African American **(B)** older adults with or without history of other psychiatric disorders (i.e., post-traumatic stress disorder (PTSD), bipolar disorder, schizophrenia, anxiety, obsessive-compulsive disorder (OCD), and developmental neuropsychiatric disorders). Models were adjusted for participants’ age at baseline, gender, education level, and BMI. Results suggest that having history of other psychiatric disorders increases the risk of developing Vascular Dementia.

We then conducted Models 1.3 (Table 2) and 2.3 (Table 3) for participants in each racial/ethnic group, respectively. Significant associations were found only in non-Hispanic White people and African Americans (Table 4). For non-Hispanic White people, having a history of other psychiatric disorders tends to increase 27% of the hazard of developing AD (HR: 1.27, 95%CI: 1.06–1.52) and 28% for VaD (HR: 1.28, 95%CI: 1.07–1.54), respectively. For African Americans, the history of other psychiatric disorders tends to increase 116% hazard of developing AD (HR: 2.16, 95%CI: 1.24–3.76) and 108% for VaD (HR: 2.08, 95%CI: 1.22–3.54), respectively. Kaplan–Meier survival curves in Figures 1, 2 depict the difference in the impact of history of other psychiatric disorders on AD and VaD onset risk in non-Hispanic White and African American older adults.

**Table 4:** Hazard ratios of other psychiatric disorders^a^ on AD (Alzheimer’s Disease) and VaD (Vascular Dementia) risks by race/ethnicity.

	AD	VaD
	HR (95%CI)	HR (95%CI)
Non-Hispanic White person	1.27 (1.06, 1.52)*	1.28 (1.07, 1.54)**
Hispanic	1.02 (0.54, 1.92)	1.01 (0.54, 1.9)
African American	2.16 (1.24, 3.76)**	2.08 (1.22, 3.54)**
Asian	1.24 (0.4, 3.81)	1.7 (0.58, 5.01)
Others	1.19 (0.53, 2.66)	1.27 (0.58, 2.76)

This table presents the hazard ratios of the impact of a history of other psychiatric disorders on the risks of AD and VaD, as estimated from 10 Cox models stratified by race/ethnicity. The first column displays the results of the five models examining the effect of other psychiatric disorders history on AD risk. The second column displays the results of the five models assessing the effect of other psychiatric disorders history on VaD risk. ^a^Other psychiatric disorders include post-traumatic stress disorder (PTSD), bipolar disorder, schizophrenia, anxiety, obsessive–compulsive disorder (OCD), and developmental neuropsychiatric disorders. AD models were adjusted for age, sex, education, BMI, FTLD mutation, and APOE-e4 alleles. VaD models were adjusted for age, sex, education, and BMI. *p < 0.05, **p < 0.01.

## Discussion

This study investigated the impacts of psychiatric and substance disorder history on the incidence of different etiological types of dementia (i.e., AD and VaD), and examined how these effects varied across different racial/ethnic groups. Despite that many studies have examined associations between some psychiatric disorders and prevalence of dementia (29, 64, 65), evidence regarding the longitudinal effects of psychiatric disorders history on the risks of various etiological dementias remains sparse. Such research can help understand the underlying mechanisms that contribute to cognitive decline and identify intervention options for people with psychiatric disorders history. The findings of this study demonstrated that individuals with a history of psychiatric disorders (i.e., depression, other psychiatric disorders, traumatic brain injury, and alcohol use) experienced a significant increased risk of both AD and VaD over a 14-year follow-up period. Interaction analyses revealed a significant relationship between racial/ethnic identity and a history of other psychiatric disorders, suggesting that non-Hispanic White people and African Americans may be more susceptible to AD and VaD if they have a history of other psychiatric conditions.

This study confirmed findings from earlier research that a history of depression, other psychiatric disorders, TBI, and alcohol use significantly increased the risk of AD and VaD (64, 66). However, our findings reported increased risk was less than previously reported. For instance, an earlier systematic review revealed that schizophrenia and bipolar disorder increase a person’s risk of dementia by at least two-fold, whereas the history of other psychiatric disorders in this study is only associated with a 25 and 24% increase in AD and VaD risk, respectively (18, 28). Heavy use of alcohol was found to double the risk of dementia (67), whereas our findings reported a 22% increased risk. One possible reason is that the sample in this study was gathered primarily through clinic referrals, and the characterization of index diagnoses was rendered through expert consensus guidelines which may not be the case in previous studies. Thus, sample characteristics as well as specific diagnostic ascertainment may have attenuated the association between depression, other psychiatric disorders, TBI, alcohol use, and incident dementia in our analysis.

Although earlier research has shown that heavy or prolonged use of illicit substances (e.g., cannabis, opioids, etc.) is linked to a higher risk of dementia (68, 69), our findings did not support this link. This might be related to the assessment of other substances use history, which only inquired about clinical impairments in work, driving, legal, and social activities caused by other substance use in the past 12 months. Variable operationalization, such as whether to identify substance types and duration of substance use, may have an impact on the findings (68). For instance, it was found that light to moderate alcohol consumption and marijuana use during middle age are not linked to dementia risk in later-life (39, 70). Future research should employ more detailed measures to evaluate how substance use affects the risk of incident dementia.

Similar patterns were found in both sets of models for AD and VaD, indicating that a history of psychiatric disorders significantly increase the risks of both etiological dementias. This may be due to similar neuropsychiatric mechanisms that underlie the onset of these two dementias. For example, depression and schizophrenia are related to the accumulation of amyloid beta and tau proteins in brains, which are both important biomarkers of AD and important risk factors for cerebrovascular diseases (27). Additionally, excessive alcohol and drug use can reduce neural activity and raise the risk of chronic illnesses like hypertension, which, in turn, raises the risk of various dementia etiologies (66). These results highlight the importance of treating psychiatric disorders to prevent dementia onset and enhance cerebrovascular and functional health.

For the racial/ethnic disparities in dementia risk, we found that history of other psychiatric disorders is only related to the increase of dementia risk in non-Hispanic White people and African Americans, but not for Hispanic and Asian older adults. This is aligned with the Hispanic paradox in cardiovascular and cognitive health, that is, Hispanic older adults have a lower risk of cardiovascular disease and cognitive impairment than non-Hispanic White people (71). Hispanics tend to have robust cultural and familial values and social networks, making it simpler to maintain social engagement and access alternative treatment options when dealing with psychiatric disorders. Also, a bilingual living environment may have a protective effect on older Hispanic adults’ cognitive function and access to care. Similarly, many older Asian adults still maintain traditional Asian values and lifestyles, such as emphasizing family, healthy diet, and regular physical activities. They also adopt bilingualism in their daily lives (72), which similar to Hispanics, may be protective.

Notably, older African Americans had twice the risk of AD and VaD if they had a history of other psychiatric disorders. Previous research has identified a wide range of potential response for why African Americans have a higher risk of dementia than non-Hispanic White people, including higher cardiovascular disease prevalence, increased exposure to racism and stress, lower socio-economic status, and limited access to healthcare resources (73). This finding underscores the need for understanding the multiple health disparities pathways impacting brain functioning and mental health for this group as cited in the National Institute on Aging Health Disparities Framework (74), and understanding the sources of resilience (cultural, neurological, etc.).

Although we did not find interaction effects for the remaining racial/ethnic groups in our study, we acknowledge that there may be differences in how psychological and emotional symptoms are communicated to professionals, and by extension, researchers in academic settings. A robust body of literature highlights that people differ in symptom presentation and role of stigma which may affect the accuracy of diagnostic rates, which coupled with clinician’s lack of training and potential biases with regard to group-specific nuances, may affect diagnostic ascertainment rates—either lower or higher (51, 75–78).

The findings of this study have implications for future research and clinical practice. In terms of research, there is a need for further investigation into the impact of clinical treatments, both pharmaceutical and non-pharmaceutical, on the cognitive health of individuals with psychiatric and substance abuse disorders. Current prognostic measures primarily focus on preventing relapse, but patients often have low medication adherence due to the side effects of anti-psychotics (29, 64, 66). Studies have shown that taking anti-psychotic medications for over 3 months may increase the risk of all-cause dementia (79). Non-pharmaceutical interventions, such as cognitive behavioral therapy and healthy lifestyle programs, may be potential alternatives to anti-psychotics for helping people with mental illness to manage and reduce symptoms, while the effectiveness of such interventions still lacks robust randomized experimental studies to confirm (80). Further research in these areas will be essential to better understand the mechanisms of psychiatric disorders and relevant treatments on cognitive health, which can inform clinical decisions and improve health outcomes.

Moreover, it is crucial for researchers, healthcare professionals, and community service providers to be aware of the racial/ethnic differences in dementia risk as well as any potential socio-economic, educational, cultural, and linguistic barriers. For older people, it is essential to develop and deliver interventions and treatments that are culturally attuned, age-appropriate, and respectful of how brain health concepts are communicated. Additionally, more outreach and education initiatives ought to be made in communities with low socioeconomic status, limited exposure to information on brain health and psychiatric and substance use conditions, and limited access to healthcare resources.

## Limitations

We note study limitations: First, the study sample is derived from the NACC UDS, which is not representative of the US population (52). Nevertheless, the database compiles interdisciplinary data from more than 30 ADRCs in the US, and its vast data volume increases the accuracy of index disorder diagnoses, and the estimation of dementia risk. We also employed advanced analytical techniques, like multiple imputations, to minimize data loss during the analyses. The large sample size may reduce concerns about collider bias, strengthening the validity of the results. Second, the analyses divided participants’ racial/ethnic identities into five groups including an “other group,” which obscures data on health disparities between some racial/ethnic groups. For instance, different Asian ethnic groups (e.g., Chinese, Korean, and Vietnamese) may have varying dementia risk (81), and the same goes for American Indians and Alaska Natives. Third, results of this study may be impacted by the measurement of some variables and the development of analytical models. For instance, this study did not examine the relationship between tobacco abuse history and dementia risk, as the UDS data only collects participants’ current smoking behaviors, which cannot be used to determine the presence of a heavy tobacco use history. Given that heavy smoking in midlife can increase the risk of AD and VaD, and its contribution to higher dementia risk in African Americans compared to non-Hispanic White individuals (35, 82), future research should explore the interaction between tobacco use history and other psychiatric disorders to better understand the elevated dementia risk observed among African Americans with other psychiatric histories. Lastly, lifestyle behaviors, access to care, and living conditions were left out of models. Future research should examine the physiological, social, and behavioral mechanisms of dementia onset using more thorough assessment techniques and analytical frameworks.

## Conclusion

In sum, this study investigated the impacts of psychiatric disorders histories (i.e., depression, other psychiatric disorders, traumatic brain injury, alcohol use, and other substance use) on the risk of AD and VaD in older adults of different racial/ethnic groups. We found that the history of various psychiatric disorders has similar patterns in the impact on the risk of AD and VaD. And the history of psychiatric disorders has similar effects on dementia risk across different racial/ethnic groups, except for other psychiatric disorders. This study encourages further investigation into the mechanisms by which the history of psychiatric disorders affects dementia risk to help develop intervention and treatment options. Future research should address diagnostic shortcomings in the measurement of psychiatric and substance use disorders in ADRCs, especially with symptom presentation and the role of stigma. Lastly, future research and practice efforts should consider innovative clinical and public health interventions for the general population as well as targeted cognitive health strategies aligned with the needs and preferences of persons living with AD and VaD from underserved racial/ethnic communities.

## Data availability statement

Publicly available datasets were analyzed in this study. This data can be found at: https://naccdata.org.

## Ethics statement

Ethical approval/written informed consent was not required for this human studies as it uses retrospective public data.

## Author contributions

MA conceived the research question and protocol. XW cleaned the data. JL conducted the study analyses. MA and JL contributed equally to the writing and revision of the manuscript. All authors approved the final version of the manuscript.

## Funding

Funding for this work was provided by the National Institute on Aging (NIA) of the National Institutes of Health (NIH) under award number P30AG066530, and the Della Martin Foundation. MA was also funded by U54AG063546 which funds NIA Imbedded Pragmatic Alzheimer’s Disease and AD-Related Dementias Clinical Trials Collaboratory (NIA IMPACT Collaboratory), and the Minority Aging Health Economics Research Center (RCMAR) under award number P30AG043073 (NIA/NIH). The content is solely the responsibility of the authors and does not necessarily represent the official views of the National Institutes of Health.

## Conflict of interest

LS reports personal fees from AC Immune, Athira, BioVie, Eli Lilly, Lundbeck, Merck, Neurim Ltd., Novo-Nordisk, Otsuka, Roche/Genentech within the past year; and research grants from Biogen, Eisai, Eli Lilly within the past year.

The remaining authors declare that the research was conducted in the absence of any commercial or financial relationships that could be construed as a potential conflict of interest.

## Publisher’s note

All claims expressed in this article are solely those of the authors and do not necessarily represent those of their affiliated organizations, or those of the publisher, the editors and the reviewers. Any product that may be evaluated in this article, or claim that may be made by its manufacturer, is not guaranteed or endorsed by the publisher.

## Supplementary material

The Supplementary material for this article can be found online at: https://www.frontiersin.org/articles/10.3389/fpsyt.2023.1165262/full#supplementary-material
