# Supplementary material for: The relationship of history of psychiatric and substance use disorders on risk of dementia among racial and ethnic groups in the United States
Source: Front Psychiatry. 2023 Apr 24;14:1165262. doi: 10.3389/fpsyt.2023.1165262 (PMC10165105; doi:10.3389/fpsyt.2023.1165262)
Supplement: Supplementary file 1 [file Table_1.DOCX]

Supplementary Table 1. Sensitivity analyses that compare explanation power and model fit between competing risk models and Cox hazard models

|  | | | Competing risk models | Cox hazard models |
| --- | --- | --- | --- | --- |
| AD | Any psychiatric disorders | c-index | 0.684 | 0.692 |
|  |  | AIC | 48347 | 46551 |
|  |  | BIC | 48354 | 46660 |
|  | Depression | c-index | 0.684 | 0.692 |
|  |  | AIC | 48365 | 46541 |
|  |  | BIC | 48372 | 46650 |
|  | Other psychiatric disorders | c-index | 0.673 | 0.687 |
|  |  | AIC | 48411 | 46612 |
|  |  | BIC | 48418 | 46721 |
|  | Traumatic brain injury | c-index | 0.674 | 0.687 |
|  |  | AIC | 48391 | 46613 |
|  |  | BIC | 48399 | 46722 |
|  | Alcohol abuse | c-index | 0.671 | 0.685 |
|  |  | AIC | 48416 | 46621 |
|  |  | BIC | 48423 | 46730 |
|  | Other substance abuse | c-index | 0.671 | 0.686 |
|  |  | AIC | 48423 | 46624 |
|  |  | BIC | 48431 | 46733 |
|  | | | | |
| VaD | Any psychiatric disorders | c-index | 0.608 | 0.704 |
|  |  | AIC | 5657 | 5330 |
|  |  | BIC | 5665 | 5408 |
|  | Depression | c-index | 0.605 | 0.702 |
|  |  | AIC | 5666 | 5334 |
|  |  | BIC | 5674 | 5412 |
|  | Other psychiatric disorders | c-index | 0.652 | 0.676 |
|  |  | AIC | 5684 | 5372 |
|  |  | BIC | 5692 | 5450 |
|  | Traumatic brain injury | c-index | 0.656 | 0.678 |
|  |  | AIC | 5685 | 5374 |
|  |  | BIC | 5693 | 5452 |
|  | Alcohol abuse | c-index | 0.664 | 0.682 |
|  |  | AIC | 5678 | 5370 |
|  |  | BIC | 5698 | 5448 |
|  | Other substance abuse | c-index | 0.652 | 0.676 |
|  |  | AIC | 5682 | 5370 |
|  |  | BIC | 5690 | 5448 |
