# Supplementary material for: The relationship of history of psychiatric and substance use disorders on risk of dementia among racial and ethnic groups in the United States
Source: Front Psychiatry. 2023 Apr 24;14:1165262. doi: 10.3389/fpsyt.2023.1165262 (PMC10165105; doi:10.3389/fpsyt.2023.1165262)
Supplement: Supplementary file 2 [file Table_2.DOCX]

Supplementary Table 2. P values of all LR tests that compared Cox models with/without interactions

|  | AD | VaD |
| --- | --- | --- |
| Any psychiatric disorders | 0.096 | 0.087 |
| Depression | 0.074 | 0.073 |
| Traumatic brain injury | 0.084 | 0.434 |
| Alcohol abuse | 0.054 | 0.127 |
| Other substance abuse | 0.161 | 0.216 |

Notes: This table presents the p values when comparing models with and without the interaction term (e.g., depression history X race/ethnicity). All p values are greater than 0.05, indicating that the impacts of the history of psychiatric and substance use disorders on the risks of AD and VaD do not vary by race/ethnicity.
